# Conceptions of clinical learning among stakeholders involved in undergraduate nursing education: a phenomenographic study

**DOI:** 10.1186/s12909-021-02939-7

**Published:** 2021-10-04

**Authors:** Malou Stoffels, Stephanie M. E. van der Burgt, Terese Stenfors, Hester E. M. Daelmans, Saskia M. Peerdeman, Rashmi A. Kusurkar

**Affiliations:** 1 Faculty of Medicine, Research in Education, Amsterdam UMC, Vrije Universiteit Amsterdam, Amsterdam, The Netherlands; 2 Amsterdam UMC, Amstel Academy, Institute for Education and Training, Amsterdam, The Netherlands; 3 LEARN! research institute for learning and education. Faculty of Psychology and Education, VU University Amsterdam, Amsterdam, The Netherlands; 4 AmsterdamUMC, Location AMC, Institute for education and training, Amsterdam, The Netherlands; 5 Karolinska Institutet, Department of Learning, Informatics, Management and Ethics, Amsterdam, The Netherlands; 6 Faculty of Medicine, Department of skills training, Amsterdam UMC, Vrije Universiteit Amsterdam, Amsterdam, The Netherlands; 7 Department of Neurosurgery, Amsterdam University Medical Center, Vrije Universiteit Amsterdam, Amsterdam, The Netherlands

**Keywords:** Clinical learning, Nursing education, Phenomenography, Student engagement

## Abstract

**Background:**

To prepare nursing students to become critical, autonomous members of the workforce, an agreement among stakeholders on how this can be achieved in the clinical setting is needed. However, a critical discussion of the clinical learning process in relation to actual and desirable outcomes is lacking in the nursing education literature. This study aimed to map conceptions of the desired process and outcomes of clinical learning among stakeholders involved in undergraduate clinical nursing education.

**Methods:**

Twenty-five semi-structured interviews about their understanding of clinical learning were conducted with nursing students, supervisors, clinical educators and higher education institute professionals involved in clinical nursing education in a Dutch academic medical center. Data were analyzed using a phenomenographic approach.

**Results:**

Four conceptions were identified: clinical learning as a process to 1) meet curricular demands, 2) learn to deliberately deliver patient care, 3) learn to deliver patient care within the larger (healthcare) context, and 4) become a continuously developing professional. Conceptions 3 and 4 represented a broader, more inclusive perspective on clinical learning than conception 1 and 2. Conceptions were distinguished by five dimensions: role of guidelines from the school; learning opportunities, focus of supervisor; focus of reflection; desirable outcomes of clinical learning.

**Conclusions:**

Those directly involved in clinical learning in nursing may have qualitatively different understandings of its desired nature and outcomes. Two patterns across conceptions could be discerned: a) a shift in focus from learning as following standards, to following an individualized learning trajectory and b) a shift in focus from increasing patient load, to understanding oneself and the patient within the healthcare context. To prepare nursing students for the future workforce, a flexible, social form of self-regulated learning is warranted, as well as an understanding of one’s own role within the healthcare system and a critical attitude towards healthcare. Schools and hospitals should collaborate to integrate these values in the curriculum. The current study adds different ways of applying self-regulated learning as a relevant dimension in understanding clinical learning to the literature. Through the phenomenographic approach we identified conceptions that can be a basis for training and policy development.

**Supplementary Information:**

The online version contains supplementary material available at 10.1186/s12909-021-02939-7.

## Background

In the past decades, undergraduate nursing education has shifted from an apprentice-based training model to institution-based programs in response to the increasing complexity of the profession [1]. However, suboptimal outcomes in terms of students’ critical thinking and readiness for independent practice are still reported [2]. Despite the development of theory in nursing education, clinical education is often provided in ad hoc ways [1, 3]. A critical discussion of the clinical learning process in relation to actual and desirable outcomes is lacking in the nursing education literature [3, 4]. Therefore, we explored conceptions of clinical learning in the hospital setting among nursing students, supervisors, clinical educators and higher education professionals. Articulating and discussing these conceptions can help to improve clinical learning at the policy making level as well as in practice [5].

In clinical nursing education, students learn through delivering patient care, in interaction with staff and peers, supported by reflection [4]. The academic and clinical setting collaborate to strengthen students’ clinical learning through supervisor training, tools for students to support reflection and active learning, the presence of school teachers in practice and vice versa, and organizational models to support independent working and peer learning [6–8]. However, individual students and supervisors have different interpretations and applications of tools to structure learning such as learning goals [9]. Moreover, the literature reports several aims students have to reconcile when engaging in clinical learning, such as contributing to patient care [1, 10], belonging to the team [11], and meeting assessment standards [12], resulting in different learning preferences, behavior and outcomes. Hereby, the individual learning process and outcomes remain dependent on individual students’ and staff engagement [13].

The study of conceptions of learning and teaching can help understand differences in preferences and behavior [14]. In medical clerkships, it was found that supervisor’s conceptions of workplace learning varied in the extent to which student’s learning was regarded as learning as a way to acquire knowledge or a way to participate in the community [15]. In nursing education, supervisors have different understandings of nursing itself as well as their role in facilitating student learning [16, 17]. Recently, a study on nursing students’ conceptions of clinical competence and the learning process was published, identifying conceptions of competence ranging from task completion to yielding positive health outcomes [18]. The current study adds to this work by extending it to a different setting, and by including a broader set of potential ‘outcomes’ of clinical learning than ‘competence’ only. Moreover, we included conceptions of other direct stakeholders than students only.

Clinical learning in nursing education has been studied from different theoretical perspectives, often falling under the umbrella of experiential learning theories [4]. These constructivist theories share the notion of learning as an individualized, active trajectory in an authentic environment, shaped by social interactions and reflection [4, 19]. Surprisingly, in studies on clinical learning in nursing education, little reference is made to how clinical learning is affected by the formal curriculum, suggesting a hard separation between ‘formal’ academic learning and ‘informal’ clinical learning [4]. Clinical nursing education involves a three-way partnership between higher education institutes, students, and clinical placement settings such as hospitals. Two theoretical perspectives are particularly relevant for studying how learning within this context can be maximised. Firstly, cooperative education, also labeled work-integrated learning, which is a strategy of education combining academic learning with real-world practice in a relevant workplace [20]. Leaning on theories of experiential learning, reflective practice, and sociocultural views of learning, this perspective suggests that the integration of classroom and practice experiences is crucial to maximise student learning. Secondly, self-regulated learning (SRL) which is a cyclical process of seeking to accomplish goals through the self-directed use and modification of strategies [21–23]. Educational institutes play a role in offering students tools to maximize clinical learning by strengthening their self-regulating learning process [24].

Thus, clinical learning can be strengthened within the collaboration between the clinical and academic settings, however, how to put this to practice in day-to-day clinical learning is not straightforward. Therefore, we asked the following research question:

*What different conceptions do stakeholders involved in undergraduate nursing education have of the desired process and outcomes of clinical learning?*

We choose a phenomenographic approach as this would allow us to distinguish different ways of experiencing the same phenomenon, in this case clinical learning [25]. In line with experiential learning theories, all intended and unintended activities and outcomes that students experience during their clinical placements were considered as ‘clinical learning’ [19]. We preferred the broad concepts ‘clinical learning’ and ‘outcomes’ to concepts ‘cooperative education’, ‘self-regulated learning’ or ‘competence’ to stay as close as possible to stakeholders personal experiences and frames of reference. In order to gain an in-depth understanding of the breadth of conceptions within this group of stakeholders, we did not consider potential differences between subgroups involved in clinical learning.

## Method

### Background of the methodology

Phenomenography is a research method to answer questions about how people experience the world around them, originally developed in the field of teaching and learning [30, 32]. Phenomenography is based on the assumption that there are limited qualitatively ways in which a phenomenon can be experienced, conceptualized and understood [25]. Phenomenography is distinct from phenomenology. While *phenomenology* investigates the essence of lived experiences of a phenomenon, *phenomenography* considers the variation of ways of understanding a phenomenon [33].

To describe this variation, different *ways of understanding* a phenomenon (constructed from an entire pool of data) as opposed to *different individual conceptions* are the main outcome of phenomenography. These ways of understanding are called ‘categories of description’ [30]. These abstracted conceptions cannot be attributed to individuals and individuals can carry conceptions that fall into different categories [34, 35]. However, categories of description can inform us about the variety of possible conceptions. Therefore, they will be referred to as ‘conceptions’ throughout the paper.

In addition to conceptions, relationships between conceptions are identified. These relationships are represented in a table called an ‘outcome space’, showing how conceptions vary on underlying ‘dimensions of variation’. Dimensions of variation are aspects of a phenomenon discerned by participants that distinguish conceptions from each other [35]. Conceptions are often hierarchically related, representing an expanding understanding of underlying dimensions [29, 31, 35, 36].

### Setting

We conducted this study in an academic medical center in the Netherlands. This hospital offers clinical nursing placements (5–40 weeks) for two education tracks, one at the vocational and the other at the higher education level, for which theoretical training is provided by several vocational and higher education partners. These different education tracks are both 4-year programs with a minimum of 2300 practice hours, resulting in the same job title. In both tracks, students work towards competency achievement on the basis of individual learning plans and learning goals, thus (implicitly) embracing principles of self-regulated learning [22]. Assessment procedures and criteria differ between higher education institutes and involve a combination of CanMEDs, patient load and patient complexity. Practice curricula are designed in collaboration between higher education institute policy makers/ teachers and clinical teachers/supervisors. In clinical practice, students work alongside a clinical supervisor, who is a registered nurse with basic training in supervisory skills such as giving feedback. Additionally, each nursing ward has a clinical educator, who is exempted from patient care but coordinates clinical training and coaches students and clinical supervisors. Typically, students sit together with their supervisor at the start and the end of the day to discuss learning goals and evaluate learning. Some of the nursing wards are modelled as ‘dedicated educational units’ [26, 27].

### Participants and sampling

The secretary of the clinical nursing education department sent an information and recruitment email to all stakeholders in undergraduate clinical learning in our hospital in April 2019. Stakeholders were defined as those directly involved in clinical learning, and we invited 123 students, 177 clinical supervisors, 12 clinical educators and 12 higher education institute professionals (nursing teachers that are involved in mentoring and assessing students during clinical placements and offer classes or meetings around the ward) to participate. Stakeholders interested in participating were asked to provide the following background information required for sampling: age, current ward, educational institution, study track and year – only for students, position in the last two years – only for higher education institute professionals.

In phenomenography, data are treated as a holistic group, and not as a set of individual interviews. Therefore typically one round of data collection and one type of data is used, and the number of participants is determined in advance [28]. The number of participants in phenomenographic studies usually ranges between 10 and 30 with maximum variation within the sample, to capture the various ways in which a phenomenon is understood and to keep data manageable [29]. Given our diverse population consisting of different subpopulations, we decided to recruit as many as 25 participants. From 39 respondents, a sample of 25 participants was invited to participate in the study. To establish maximum variation, we selected our sample based on a) representation of subgroups accounting for their actual size and b) maximum variation within subgroups based on (demographic) characteristics provided..

### Data collection

We used individual semi-structured interviews to collect our data [30], as the point of departure of phenomenography is the individual’s relationship with a phenomenon [25]. MS and SEMB conducted two pilot interviews with colleagues from the broader research team to adjust the interview protocol and align the interview styles. Data from these interviews were not included in the analysis.

MS and SEMB carried out audio-recorded interviews (averaging one hour) in a private room within the hospital or the higher education institute, between April and September 2019. MS conducted interviews with students, supervisors and higher education institute professionals. To prevent any conflict of interest SEMB conducted interviews with clinical educators, with whom MS had the possibility of professional collaboration outside this research project.

We used an interview guide (see additional file 1), consisting of the main questions to describe a recent successful and less successful student’s shift in clinical practice. We asked open follow up questions to specify the information reported in as much detail as possible and to explore the interviewees’ intentions behind bringing up points in a certain way [31]. This approach allowed us to use concrete experiences as a starting point for discussing underlying conceptions. We encouraged participants to answer the questions in an in-depth manner.

### Analysis

A research assistant transcribed audio recordings of the interviews verbatim. We analyzed data along seven steps described in the literature (Table 1) resulting in an outcome space of ‘conceptions’ (ways of understanding the phenomenon of clinical learning) and ‘dimensions of variation’ [35]. The approach did not aim or allow for comparison between subgroups. During the analysis process, we found that several statements were made by all stakeholders. Moreover, we found that particular practical challenges and conflicts concerning learning in practice were reported recurrently. As these challenges and conflicts provide a useful context for the interpretation of conceptions, we decided to add these to our results. Therefore, from step 3, we kept a separate record with a) statements that reflected an *underlying shared understanding* because they were shared amongst all stakeholders and were not distinctive of conceptions, and b) statements that reflected recurrent *practical challenges or conflicts* in learning in practice. We used Atlas.ti software to code and retrieve data.

**Table 1:** Seven steps of analysis in phenomenography, with elaborations for this particular study

Step	Description [33]	Procedure in this particular study *Note: Criteria to distinguish dimensions and conceptions were: a) being qualitatively distinctive. and b) covering the whole variety of meaning units* [36].
1 Familiarization	Reading all transcripts	• All transcripts by MS • 5 transcripts by SEMB
2 Condensation	Identifying and coding meaningful units in the transcripts	• Coding of 5 transcripts by MS and SEMB • Discussion between MS and SEMB • Agreement on codes to be used (dimensions of variation in the transcripts) • After agreement, coding of rest of the transcripts by MS • Note taking by MS of different ways of understanding clinical learning across codes
3 Comparison	Comparing the units with regard to similarities and differences	Performed iteratively: • Refining dimensions of variation by looking for differences and similarities within codes by MS • Grouping similar ways of understanding clinical learning across codes in conceptions by MS • Looking for structural relationships between categories and dimensions and creating a draft outcome space of conceptions (rows) and dimensions of variation (columns) • Discussion and refinement of outcome space including labelling and hierarchy between MS and SEMB until consensus was reached • Discussion of outcome space, labels and hierarchy of categories within the research team until consensus was reached
4 Grouping	Allocating answers expressing similar ways of understanding the phenomenon to the same conceptions	
5 Articulating	Capturing the essential meaning of a certain conception	
6 Labelling	Expressing the core meaning of the conception	
7 Contrasting	Comparison of conceptions with regard to similarities and differences	

### Reflexivity

We established rigor by following the steps of the phenomenographic approach [28, 31]. The study was reported according to the COREQ checklist [37] (see additional file 2). A research team that was diverse in background and occupation limited personal or disciplinary bias [38]. MS is trained in psychology and education and works as an educational consultant and researcher in nursing education, SMEB is trained in sociology and works as a researcher in postgraduate medical education, TS is trained in social sciences and works as a professor in medical education with expertise in phenomenography, HEMD is trained in medicine and works as a head of the master’s program in medicine, SMP works as a neurosurgeon, professor in education and vice dean, RAK is trained as a medical doctor and works as an associate professor in medical education. All authors are trained and have experience in qualitative research. MS was the only one who was involved in a professional (non-hierarchical) relationship with some of the participants. To overcome a possible conflict of interest, in such cases the interviews were conducted by SEMB. In each refinement of the outcome space, we revisited the transcripts. We enhanced credibility of the analyses through group discussions aiming to look for disconfirming evidence and to challenge findings with reference to transcripts. We kept notes from all the steps; MS and TS repeatedly discussed progress and doubts together with the draft results.

## Results

### Participants

We interviewed 25 participants including 8 students, 4 higher education institute professionals, 9 supervisors and 4 clinical educators. Students, supervisors, and clinical educators were distributed over 13 different nursing wards (hematology, cardiology, obstetrics, medical, medical psychiatry, internal medicine, neurology, Ear, nose & throat, short stay, traumatology, pulmonology, vascular surgery, oncological surgery, acute admissions ward). Demographic characteristics are presented in Table 2.

**Table 2:** Demographics of participants

Subgroup	Age (in years)	Gender
Students	Range 20–42 Mean 26 Median 24.5 (study year 2–4)	1 M; 7 F
Supervisors	Range 24–51 Mean 34 Median 29	0 M; 9 F
Clinical educators	28–64 Mean 43 Median 39.5	0 M; 4 F
Higher education professionals	Range 30–58 Mean 48.25 Median 52.5	2 M; 2 F

### Main findings

#### Conceptions

Four qualitatively different conceptions of the desired process and outcome of clinical learning were identified, which are described in more detail below.

Clinical learning as a process to …

1. Meet curricular demands
2. Learn to deliberately deliver patient care
3. Learn to deliver patient care within the larger (healthcare) context
4. Become a continuously developing professional.

#### Relations between conceptions

Five dimensions of variation were created from the data that distinguish the different conceptions: *role of guidelines from the school; learning opportunities, focus of supervisor; focus of reflection; desirable outcomes of clinical learning.* The relations between the four conceptions and the five dimensions of variation are mapped in an outcome space, see Table 3.

**Table 3:** Outcome space

		Dimensions of variation				
		Role of guidelines from the school	Learning opportunities	Focus supervisor	Focus of reflection	Desirable outcomes of clinical learning
Conceptions	**1.Meet curricular demands**	Provide directions (Prescriptive)	Planned situations matching students’ competency level based on their learning goals	Correct, monitor, give feedback to predetermined learning goals	Collect evidence with predetermined formats	Student has completed (personal) learning goals /can independently care for a certain number of patients
	**2. Learn to deliberately deliver patient care**	Provide directions *which should be interpreted within the context*	Both planned and unplanned situations, *as long as they are reflected on*	Respond to predetermined learning goals *and actively stimulate student*	Supported by formats and focused on improvement, *With input from supervisor; deliberately, linked to actual clinical events*	In addition to column 1: *Student has a better understanding of individual patient care and developed their own ways of working*
	**3. Learn to deliver patient care within the larger (healthcare) context**	Provide rough directions *but can be limiting*	Both planned and unplanned situations, *including those forcing to look beyond the individual patient-nurse relationship*	Respond to predetermined learning goals, actively stimulate students *and challenge them to look beyond the individual patient-nurse relationship*	Supported by formats and focused on learning, *With input from patient and peers; in peer/reflection meetings; beyond individual patient*	In addition to columns 1 and 2: *Student has become aware and critical of his/her role within the chain, and the patient within the healthcare system*
	**4. Become a continuously developing professional**	Provide rough directions *for future development*	Both planned and unplanned situations, *with a focus on those that are unexpected and make students stretch their boundaries*	Respond to predetermined learning goals, actively stimulate students *and challenge them to develop their own learning process*	Supported by format of choice, *Personalized; in a way and modality that suits student*	In addition to columns 1 and 2: *student can create new learning opportunities and ways to grow from them as a nurse, stretched personal boundaries*

The relationships between dimensions of variation in the outcome space showed a branched hierarchy [34]:

- conception 2 represented a broader understanding of all dimensions of variation than conception 1
- conceptions 3 and 4 both represented a broader understanding of all dimensions of variation than conceptions 1 and 2. Conceptions 3 and 4 were equally broad, but qualitatively different.

For example, in conception 1 (meet curricular demands) the dimension ‘reflection’ is considered something happening according to fixed standards, whereas in conception 4, several possible ways of reflection (including with fixed standards) are emphasized. The part of each dimensions that distinguishes a conception from the formal ones, is written in italics.

#### Conceptions

##### Conception 1: meet curricular demands

The first conception has a task-oriented approach in which the focus of learning is a set of plannable activities that result in predetermined outcomes. The learning outcomes to be pursued are clear quantifiable requirements:

*“[requirements should be clear] because we both want to achieve the same thing. That is, that the student can care for a patient with a certain condition after so many weeks...Well, [if there were no guidelines] the danger is that everyone will apply their own guidelines, eh.. ...” (participant 22).*

These requirements in turn are the basis for creating and selecting learning opportunities:

*“If you were to do it very neatly, you would have a kind of train timetable from the NS [National Railways], which states: in week 1, day 1, I will follow xx to see how a patient is washed. In week 2 I will wash a patient with aphasia under supervision, for example, under the supervision of so and so.” (participant 22).*

The supervisor’s role is understood as to guide and protect students’ process to achieve those goals when necessary, while the student remains the main responsible to guard the process.

*“Well the student is here to achieve something, she has to meet certain competencies. Be able to demonstrate that he or she has worked on a certain learning objective in order to achieve a certain core competency. Then I think yes I would like... look it is of course the responsibility of the student himself. But I would like to be able to contribute to the fact that someone learns something when I provide supervision” (participant 11).*

The goals that the student put forward is the main source of feedback:

*Yes of course [I have to formulate learning goals] because otherwise they [supervisors] don’t know what to look at. (participant 3).*

Learning outcomes are often approached quantitatively, such as the number of patients a student can take responsibility for.

“[ I learned] *That there’s actually a lot I can do myself. That I actually …*. *like now 3 patients who are fairly low in care … that that is sometimes not enough. I think that’s really a ... yes something I realize then...”(participant 2).*

Reflection happens according to fixed standards. An important goal of writing reflections is to collect ‘evidence’:

*“Well for them it is of course a piece of evidence that they really worked on a certain learning objective and yes, that I indicate at what level and with what complexity they worked and whether that went well, yes or no. ..and if things are not going well, for example, it is also good for a work supervisor to have in black or white that things are not going well” (participant 11).*

##### Conception 2: learn to deliberately deliver patient care

In the second conception, the focus is broadened to learning that can occur anytime and anywhere. Learning outcomes depend on the extent to which the student deliberately reflects on situations.

*“Well I .... I think they always learn. If they do this well then it is fine, and if they run away and do other work, they learn from that too. Then they learn how to respond to such a situation, and then they can always figure out how they will approach it differently next time” (participant 18).*

The supervisor’s role is understood as challenging students to engage in reflection. Furthermore, the supervisor actively creates and highlights learning opportunities, both during clinical duty and off it, that may go beyond pre-established learning goals.

*“I’m always like, if you don’t know that yet, we will go and do it together. Even if you are not yet allowed to do that according to the school, then I will do it or you will have a look, you know, so that they are included in technical nursing activities, or you can learn something from everything..” (participant 13).*

The aim of these reflections is to get a better understanding of what care would be delivered in what way to individual patients.

*“Or if I say, for example: I want to pay extra attention to fluid intake, they ask me to explain that so I think about why I’m doing something instead of thinking yes, I’m just going to keep fluid balance just because I do. That I really think about why I do it” (participant 7).*

##### Conception 3: learn to deliver patient care within the larger (healthcare) context

This conception includes a broader view on what it means to be a good nurse. From the start of clinical learning, the importance of regarding the patient as part of a larger personal and healthcare context is acknowledged, and situations in which students can reflect on this are highly valued, as well as off-time to immerse oneself in patient cases and underlying theory.

*“So I think ... often the nurse is asked what is the best thing to do, but then I think: you can also ask the patient because they can also answer, they can explain it very well because they have been living with the problem for 40 years. So I think these are also nice learning moments”. (participant 12).*

*“It is important that students] engage in conversations with several disciplines about their profession, and about how to include them in a treatment plan”* (*participant 14*).

*“I would also have liked to delve much more into which part where the hospital takes within this chain care. Because that patient is not just here, he has been to the doctor, and he has home care and he has that care and he continues”*. (*participant 5*).

Likewise, learning to become a nurse is not only considered as an individual path of developing knowledge and skills, but also as a way to find one’s role within a team and an institution.

*“[when working a couple of consecutive day shifts] I think you can better realize what you are actually doing as a colleague or as a team. Even if it is mono-disciplinary. You are a relatively small part anyway, but then I do not notice what is happening to those people, and then also build a bond with the people. …*” *(participant 8).*

*“Students think it’s normal to walk around in nursing scrubs. While you may wonder, well, that’s pretty amazing you have to, why? … Well, that kind of thing, that informal: how are you going to function as ... now as a student, and later as a qualified nurse, in such an organization, in such a team. Yes, that is not directly in the curriculum, but that is very important to learn that” (participant 22).*

Experiencing different supervision styles contributes to students’ development.

*“Well I find it very valuable to see many different work styles. And I also find it interesting because I often ask dude, why do you do it like that, because I learned it so and so from that other supervisor. And I like to create my own style based on that” (participant 6).*

Part of this learning process is to develop a critical perspective on healthcare itself, which requires thinking, reading and discussing in addition to delivering healthcare.

*“And then you expect them to think like, what is the basis for this treatment plan, what is the basis for this protocol, is there a scientific basis underneath” (participant 25).*

##### Conception 4: become a continuously developing professional

In the final conception, the personalized way in which students learn and become lifelong learners is emphasized. Therefore, insight into one’s own development and challenging one’s personal boundaries is considered more important than whether this development evolves according to planning.

*“and of course, there are also people who want an 8 or a 10 and are disappointed when they have a 6. But I think what they get here, where they actually have to override their own limits, that this might be even bigger for them.” (participant 18).*

In this conception it is assumed important to recognize and follow ones’ own learning preferences.

*“There are different levels of reflection. During the shift I check: is my planning all right, is everything going according to plan, do I know what I am doing, do I have to read the protocol, do I have enough knowledge about this. You do that all the time, but then you also start thinking about less practical things: how did I deal with that patient, how did I work together with my colleagues. And that is on a completely different level, which is much more useful to me. … But if you really want to grow as a nurse as a person, you have to reflect on that other level. I think that’s what you do after the shift.” (participant 5)*.

Supervisors challenge students not only to come up with learning goals, but to express *why* they want to learn certain things.

“*Sometimes they come up with the same learning goals a few weeks in a row and then I wonder whether they have learned it or not, what is the purpose of this learning goal. Why do you still want to use this as a learning goal now”. (participant 12).*

The eventual goal of learning is to develop oneself, when necessary, *beyond* what they can learn from their role models:

*“I think that’s, especially in the nursing profession, I think it should be more focused on that [the ‘higher up’ CanMEDS roles]. Because you can tell that most nurses who now work on the ward do not have much experience with that at all. They haven’t heard from EBP* [Evidence Based Practice, MS] *or anything. They don’t know all that, they don’t care much about them either. So it is still difficult to get supervision on those topics” (participant 14).*

#### Underlying shared understanding and structural challenges and conflicts

In phenomenography, conceptions are described by their differences, rather than commonalities [35]. However, as these commonalities can provide a context to interpret these conceptions, we decided to include them in our analysis and label them ‘shared understanding’. A safe environment characterized by trust, acceptance and sensitivity for students’ well-being in which they can gain self-confidence was considered a prerequisite for learning. Moreover, students learn by steadily gaining autonomy in both clinical work and their learning process.

Moreover, we identified practical challenges and conflicts that were reported structurally. These may reveal barriers to acting according to conceptions, hereby informing implications of the conceptions which we found. They include insecurity about responsibilities, and (perceived) burdens for students to follow their individual development pace, such as being in a subordinate position. Moreover, challenges in establishing mutual trust between students and supervisors were found, such as receiving freedom without feeling lost, and receiving supervision without feeling controlled. Both shared understanding and structural challenges and conflicts are summarized in Table 4.

**Table 4:** Underlying shared understanding and structural challenges and conflicts

	Shared understanding	Practical challenges and conflicts
**School guidelines**		- Lack of clarity about who’s responsibility it is to carry out and know school regulations
**Learning opportunities**	- When necessary, priority of patient care over learning - Students learn by a process of graded autonomy - Students should take increasing responsibility in their learning process	- The subordinate and temporary position of students hinders them in their freedom to develop in their own way
**Focus supervisor**	- Relationship of mutual trust, acknowledgement and expressed expectations between student and supervisor is essential - Boosting students’ confidence creates ‘space’ to learn.	Supervisors face a few challenges: - To balance between being supportive yet giving responsibility - to balance between giving constructive (corrective) feedback and promote safety - to practice supervision within time and training constraints - to balance between ‘challenging students to think and ‘questioning/testing students’
**Desirable outcomes of clinical learning**	- The subjective experience of clinical learning is important, to have ‘a good feeling’ about a shift	- It is challenging to develop a critical attitude while in training - Following individual pace can be at odds with assessment standards
**Peers**	- Peers are sources of help and support and mutual learning, yet can be competitors	

## Discussion

### Summary of findings

Our study shows that those directly involved in clinical learning in undergraduate nursing education may have qualitatively different understandings of its desired nature and outcomes. This variety exists in spite of a close collaboration between higher education institutes and hospitals to align clinical learning with the formal curriculum. Two patterns across conceptions can be discerned: the narrowest conception understands clinical learning as following rules and standards, whereas conceptions that are more inclusive regard clinical learning as an individualized learning trajectory with multiple possible outcomes. Second, the narrowest conception focuses on increasing individual patient load, whereas conceptions that are more inclusive aim to understand oneself and the patient within the healthcare context. Conceptions exist on top of a shared understanding that students should learn in a safe environment, with progressive independence over both their learning process and patient care. Moreover, we identified practical challenges and conflicts that may reveal barriers to acting according to conceptions.

### Contribution to previous literature on conceptions of clinical learning

The shift in focus from external standards, to more personalized critical learning aligns with previous work on nursing students’ and medical supervisors’ conceptions [15, 18]. The current study adds different ways of applying self-regulated learning as a relevant dimension in understanding clinical learning, possibly reflecting the values of modern nursing curricula. Interestingly, the shift in focus from individual nursing performance to team performance was found in conceptions of medical supervisors, but not of nursing students [15, 18]. In addition to previous literature, our focus on the broad concept of ‘outcomes of clinical learning’ gave us insight in how individuals integrate different aims such as learning, performing nursing care and meeting assessment standards to guide their behavior. Through the specific research approach used in our study, we elaborated conceptions that can help bridge between discourses of education and healthcare settings and can be the basis for training [5, 39, 40].

### Strengthening clinical learning by students’ active engagement in their learning process

In line with previous literature, respondents agree that students are supposed to demonstrate progressive independence in setting and monitoring learning goals [24]. However, in previous literature on conceptions of clinical learning, self-regulated learning was only articulated in the most inclusive conceptions [18]. In the current study, conceptions vary in their qualitative interpretation of self-regulated learning, i.e. how flexible learning goals are created and applied. One way to interpret this variance is from a developmental perspective: In previous literature it was found that novices tend to hold on to objectives as ‘external rules’ whereas experts prefer objectives as rough personal guidelines [41]. In the current study, statements belonging to conceptions that are more inclusive are not exclusively held by expert participants nor are they claimed to apply to more advanced students only, suggesting that these conceptions cannot be simply understood as stages of development. Moreover, a fixed approach to learning goals can be problematic for both novice and expert students. First, to prepare students for lifelong learning, a flexible, opportunistic variant of self-regulated learning is required [42]. Second, in the most ‘narrow’ conceptions, the supervisor’s role is understood as being reactive to the learning goals that students set for themselves. In this sense, self-regulated learning is regarded as an individual instead of a social process, distracting supervisors from their responsibility to guide students to get the most out of the clinical placement setting and to support students with their socialization into the nursing profession [9, 17, 20, 43, 44]. To improve students’ active engagement with learning, students and staff can collaborate to consider how the premises of self-regulated learning can be put to practice [45].

### Promoting higher order learning through cooperative education

Following the premises of cooperative education, higher education institutes provide students with tools to critically analyze nursing care and reflect on different ways of practice [1, 7].. Indeed, the notion of reflection as a key ingredient in clinical practice is shared across conceptions. However, the focus of these reflections varies. The narrowest conception approaches reflection as a way to improve direct performance. More inclusive conceptions emphasize the need to move away from the individual patient and create time and space to deepen knowledge and understanding during off time around the ward. Additionally, these conceptions promote engagement in activities that are not typically mastered and supported by the ward staff such as evidence-based practice. Therefore, the current findings suggest that as long as learning and assessment focus on increasing patient load, true critical reflection will not be achieved. Therefore, schools and practice settings should collaborate to educate both students and staff about the value and application of ‘higher order nursing competencies’ on the ward to integrate more aspects of theory to practice [20, 46, 47]. As was highlighted as a practical challenge, students should be encouraged to develop a critical attitude without having to fear their role in the team or their assessment. This requires a safe learning climate as well as positive role models [17, 44, 48].

### Limitations

This study has some limitations inherent to methodological choices made. First, oral expressions that may be dependent on the context, situation and individual, have been used to make interpretations about abstract conceptions that do not represent individual respondents [49, 50]. However, the nature of the interviews asking *why* participants brought up their stories, allowed us to generate an in-depth understanding of underlying conceptions. In extracting abstract representations, the main researcher could not step aside from her own experience and involvement in clinical nursing education. However, understanding the subject may have allowed gaining deeper understanding, which was constantly checked by the research team. The sample drawn from a single academic setting as well as voluntary participation of participants may have resulted in a sample that is particularly concerned with clinical learning [51]. The current conceptions and shared understanding may reflect both the values of the curricula and of the educational climate. However, the range of conceptions provides insight in the nature of variety among stakeholders and can be a basis for further studies in different settings.

## Conclusion

In spite of a collaboration between education settings and hospitals, different conceptions exist about the type of outcomes to be achieved in clinical learning as well as the learning process to reach those outcomes. The most ‘narrow’ conception limits clinical learning to a process of setting and signing off concrete learning goals aimed at increasing patient load. In conceptions that are more inclusive learning serves to understand both oneself and the patient within a larger context, and to develop oneself continually. These conceptions are represented across all groups of stakeholders. To prepare nursing students to become critical and autonomous members of the future workforce, a shift in focus from adherence to measurable demands to cooperative, self-regulated learning is warranted, as well as a shift in focus from increasing patient load to understanding healthcare and the nurse’s role within this system.

## Supplementary Information

**Additional file 1.**

**Additional file 2.**

## Data Availability

The datasets used and/or analyzed during the current study are available from the corresponding author on reasonable request.
