# Supplementary material for: Conceptions of clinical learning among stakeholders involved in undergraduate nursing education: a phenomenographic study
Source: BMC Med Educ. 2021 Oct 4;21:520. doi: 10.1186/s12909-021-02939-7 (PMC8491399; doi:10.1186/s12909-021-02939-7)
Supplement: Supplementary file 1 — Additional file 1. [file 12909_2021_2939_MOESM1_ESM.docx]

# Additional file 1. Interview guide

**Interview protocol of actors’ conceptions of clinical learning in nursing**

**Introduction**

In this interview I would like to talk to you about learning in clinical nursing placements. The ultimate goal is to analyze, based on a large group of interviews, what different conceptions exist about clinical placements and its desirable outcomes. I am curious about your personal views and experiences. There are no right or wrong answers.

**Questions (student)**

1. Can you first tell something about yourself and your work and education experience?
2. Can you describe a recent day during a clinical placement in the hospital that you thought of as a successful shift? You may not remember everything about that day, but I would like to discuss with you as much as possible about this day. *Depending on the participant’s answer, follow up questions are asked to clarify what happened on the day and how the participant experienced this?*
   1. What did you do during this day?
   2. Why did you do this?
   3. What did others do (supervisor, practical trainer, other professions, fellow students ...)
   4. What did you learn?
   5. What did you intent to learn/ was there anything else you would have liked to learn?
   6. What was your personal relationship with you supervisor/educator/ other persons you mentioned like?

*General follow up questions:*

1. 'can you tell me more about it / can you explain it in other words / why did you say that/ why do you think that is important?
2. Can you give another example (of another shift) of what you mean?
3. You said xxx, but what does that mean? Why are you talking about it in such a way?)
4. You said x and then z, how do these perspectives *relate to each other?*
5. Can you now also give a description of a less successful shift?

*(same follow up questions as with previous question)*

1. You just described a successful and a less successful shift. Can you summarize the main differences between a successful and a less successful shift in terms of learning process?
2. Can you summarize the main differences between this successful and less successful shift in terms of learning outcomes?
3. You have given me an impression, based on examples, of what you find important about learning in clinical placements. We have talked about both the process (what happens) and the outcomes (what does the student achieve). Is there anything you can add to this regarding a clinical placement *as a whole*?
4. Are there other things with regard to these two sub-topics that have not been mentioned, but that you want to say something about?

**Questions Clinical educator / higher education professional**

*Same as for other participants, with question 2 rephrased:*

1. Can you describe a recent shift of one of your students, which you thought was a successful shift for the student? You may not have seen or experienced everything about the shift yourself, but I would like to discuss the various parts of this day with you as much as possible.
   1. What did the student do this day?
   2. What did others do (supervisor, practical trainer, other professions, fellow students ...)
   3. What did you do
   4. Why did you do this?
   5. What did you hope the student would learn?
   6. What did he/she learn?
   7. Why do you think that's important?
   8. *General follow up prompts same as for students*
2. Etc.

**Questions supervisor**

*Same as for other participants, with question 2 rephrased:*

1. Can you describe a recent shift in which you have supervised a student that you thought was a successful shift for the student."
   1. What did the student do this day?
   2. What did others do (supervisor, practical trainer, other professions, fellow students ...)
   3. What did you do
   4. Why did you do this?
   5. What did you hope the student would learn?
   6. What did he/she learn?
   7. Why do you think that's important?
   8. *General follow up prompts same as for students*
